# Aggrecan Turnover in Women with Rheumatoid Arthritis Treated with TNF-α Inhibitors

**DOI:** 10.3390/jcm9051377

**Published:** 2020-05-07

**Authors:** Anna Szeremeta, Agnieszka Jura-Półtorak, Aleksandra Zoń-Giebel, Magdalena Kopeć-Mędrek, Eugeniusz Józef Kucharz, Krystyna Olczyk

**Affiliations:** 1 Department of Clinical Chemistry and Laboratory Diagnostics, Faculty of Pharmaceutical Sciences in Sosnowiec, Medical University of Silesia, Katowice, 41-200 Sosnowiec, Poland; ajura@sum.edu.pl (A.J.-P.); olczyk@sum.edu.pl (K.O.); 2 Silesian Center of Rheumatology, Rehabilitation and Prevention of Disability of Gen. Jerzy Ziętek in Ustroń, 43-450 Ustroń, Poland; azongiebel@gmail.com; 3 Department of Internal Medicine and Rheumatology, Faculty of Medical Sciences in Katowice, Medical University of Silesia, Katowice, 40-635 Katowice, Poland; magda.kopec@gazeta.pl (M.K.-M.); ekucharz@sum.edu.pl (E.J.K.)

**Keywords:** rheumatoid arthritis, tumor necrosis factor-α inhibitors, aggrecan, CS846, AGC, ADAMTS, TIMP-3

## Abstract

This study was performed to evaluate the effects of 15-month anti-tumor necrosis factor α (anti-TNF-α) therapy on the aggrecan turnover of female rheumatoid arthritis (RA) patients. Serum was obtained from healthy subjects and female RA patients treated with TNF-α inhibitors (TNFαI) in combination with methotrexate. We measured serum levels of aggrecan chondroitin sulfate 846 epitope (CS846), aggrecan fragments (AGC), disintegrin and metalloproteinase with thrombospondin motifs-4 (ADAMTS-4) and 5 (ADAMTS-5), as well as their natural inhibitor, known as tissue inhibitor of matrix metalloproteinase-3 (TIMP-3), using immunoassay methods. Serum levels of CS846, AGC, ADAMTS-4, ADAMTS-5 and TIMP-3 were higher in female patients with RA before the treatment in comparison to healthy subjects. Ratio of ADAMTS-5 to TIMP-3 was significantly higher in RA women than in controls, whereas ADAMTS-4/TIMP-3 ratio did not differ from that in controls. During the anti-TNF-α therapy, the serum levels of 846 epitope increased, whereas levels of AGC decreased in female RA patients. Furthermore, 15 months of treatment with TNFαI downregulated serum levels of both ADAMTS, without any effect on TIMP-3 levels. These changes were accompanied by significantly reduced ratios of ADAMTS to TIMP-3. According to our results, anti-TNF-α therapy has a beneficial impact on aggrecan remodeling during RA.

## 1. Introduction

**Figure 1:**
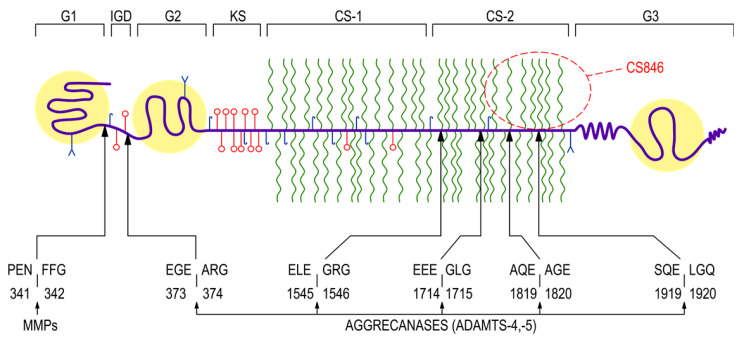
Schematic presentation of the large proteoglycan aggrecan with cleavage sites of aggrecanases and matrix metalloproteinases. Aggrecan core protein is composed of three globular regions termed G1, G2 and G3 and two extended regions forming the interglobular region located between G1 and G2 and the main region of glycosaminoglycan (GAG) attachment. The GAG-attachment region is subdivided into the keratan sulfate-rich domain and two adjacent chondroitin sulfate-rich domains (CS-1 and CS-2). The localization of aggrecan chondroitin sulfate 846 epitope is also shown, which is expressed only on the largest, newly synthesized aggrecan molecules. Amino acid sequences are written in single-letter code. ADAMTS-4 and ADAMTS-5, disintegrin and metalloproteinase with thrombospondin motifs 4 and 5; CS-1 and CS-2, chondroitin sulfate-rich domain 1 and 2; CS846, chondroitin sulfate 846 epitope; G1, G2 and G3, globular regions 1, 2 and 3; IGD, interglobular domain; KS, keratan sulfate-rich domain; MMPs, matrix metalloproteinases.

Rheumatoid arthritis (RA) is the most common chronic autoimmune inflammatory arthritis, affecting approximately 1% of the adult population worldwide. Incidence increases with age and is 2–3 times greater in women than in men. The exact cause of RA is still not completely known; however, initiation of disease seems to result from an interaction among genetic predispositions and environmental factors [1,2,3]. RA is characterized by inflammation and symmetrical joint swelling as well as progressive articular cartilage destruction and erosion of bones, with resulting fast-growing patient disability. Among mechanisms involved in joint damage, pro-inflammatory cytokines, notably tumor necrosis factor α (TNF-α) and interleukin (IL)-1β, play a crucial role in the initiation and perpetuation of the synovial inflammation and cartilage extracellular matrix (ECM) breakdown [2,3,4]. The latter one is synthesized by highly specialized mesenchymal cells called chondrocytes and is composed primarily of water molecules and type II collagen fibrous network, which entraps negatively charged proteoglycans (PGs) [4,5]. The major PG in articular cartilage is aggrecan, a large aggregating chondroitin sulfate proteoglycan, which consists of a ~250-kDa core protein with covalently attached side chains of linear glycosaminoglycans (GAGs), including approximately 100 chondroitin sulfate (CS) chains, 30 keratan sulfate (KS) chains and many N-linked and O-linked oligosaccharides [6,7,8]. The core protein of aggrecan is composed of three globular regions termed G1, G2 and G3, with a short, proteolytically sensitive, interglobular domain (IGD) separating G1 from G2 and a long GAG-attachment region separating G2 from G3 (Figure 1). The N-terminal globular G1 region is responsible for interacting with hyaluronic acid (HA) and linking protein in order to form large proteoglycan aggregates. These aggregates, characterized by high negative electrical charge, retain water molecules and endow cartilage with resistance to compression and deformation [6,7,8,9]. The G2 region has structural similarities to the G1 region but does not bind HA and its functional role is still unclear. The GAG-attachment region follows the G2 region and is organized into the KS-rich domain and two chondroitin sulfate-rich domains (CS-1 and CS-2) that differ in their amino acid composition. The G3 region resides at the C-terminal end of the aggrecan molecule and contains epidermal growth factor-like, complement regulatory and lectin-binding subdomains (Figure 1). The latter ones are able to interact with certain matrix proteins, such as tenascins or fibulins, and could potentially play a role in anchoring the aggrecan within the tissue. However, the mature aggrecan molecules are commonly devoid of the G3 region, probably as a result of proteolytic cleavage during matrix turnover [6,7,8].

During the progression of RA, extracellular matrix of cartilage is actively remodeled and the loss of aggrecan molecules is one of the earliest events which precede the breakdown of cartilage tissue [9,10,11,12]. As mentioned earlier, inflammatory factors disturb aggrecan homeostasis by decreasing its synthesis and increasing its catabolism through up-regulation of matrix degrading enzymes, such as matrix metalloproteinases (MMPs) and aggrecanases, belonging to the group of disintegrin and metalloproteinase with thrombospondin motifs (ADAMTS) enzymes [11,12,13,14,15,16]. Among the proteases involved in aggrecan catabolism, MMP-1–3, -8, -9, -13 and membrane type 1-MMP, aggrecanase 1 (ADAMTS-4) and aggrecanase 2 (ADAMTS-5) are enumerated [4,7,9,12,14,16]. The presence of these enzymes was found not only in the synovial membrane or the synovial fluid, but also in the blood plasma of RA patients [4,10,17,18]. The progressive destruction of articular structures, characteristic for RA, is not balanced by rebuilding of damaged tissues, which is caused by inhibiting effect of TNF-α and IL-1β on the formation of endogenous tissue inhibitors of these endopeptidases, i.e., tissue inhibitors of metalloproteinases (TIMPs) [13,19]. Aggrecan breakdown in cartilage matrix was ascribed to increased proteolytic cleavage of core protein. Both MMPs and ADAMTS can cleave aggrecan core protein at highly specific sites, mostly located within the IGD (Figure 1). MMPs have been shown to cleave aggrecan between the Asn^341^-Phe^342^ residues, whereas cleavage between the Glu^373^-Ala^374^ residues has been attributed to aggrecanases [9,10,11,12,16] (Figure 1). However, the ADAMTS are of particular interest because of their selectivity for aggrecan. There are also four additional aggrecanase-sensitive sites located in the C-terminal CS-2-rich region [9,12,16] (Figure 1). Overall, proteolytic cleavage of aggrecan results in generation of aggrecan fragments, which are rapidly lost into the body fluids, i.e., synovial fluid, blood and urine. It has been hypothesized that detection and quantification of released fragments of aggrecan may be useful for monitoring cartilage turnover as well as predicting disease progression in osteoarthritis (OA) and RA [4,11,20,21].

The introduction of biologic therapies based on TNF-α blockage was a revolution in the treatment of chronic inflammatory conditions such as RA. The results of clinical trials revealed that the use of TNF-α-inhibitors (TNFαI) leads to fast control of inflammation, and, most significantly, slow radiographic progression, resulting in improved physical functioning of RA patients [1,22]. However, the effect of anti-TNF-α therapy on cartilage aggrecan metabolism in RA remains unknown, which is why the main objective of this study was to evaluate quantitatively the serum biochemical markers of aggrecan turnover—i.e., ADAMTS-4 and ADAMTS-5, the proteinases responsible for aggrecan catabolism and TIMP-3, the previously mentioned proteinase tissue inhibitor—in female RA patients treated with TNF-α antagonists. Chondroitin sulfate epitope 846 (CS846) was used to evaluate aggrecan synthesis, whereas aggrecan breakdown was monitored by measuring the circulating levels of aggrecan fragments (AGC).

## 2. Materials and Methods

### 2.1. Patients and Samples

For this study, fifty female patients (mean ± standard deviation, SD) age 47.52 ± 11.91 years) meeting the 1987 or 2010 American College of Rheumatology (ACR)/European League Against Rheumatism (EULAR) classification criteria for RA [23,24] were recruited. All subjects had a Disease Activity Score 28 (DAS28) > 5.1 and did not achieve remission after the application of at least 2 synthetic disease-modifying drugs. The criteria of exclusion included prior treatment with biologic agents, withdrawal from biologic therapy during the study period, presence of illnesses (other autoimmune diseases, infections, heart failure, diabetes mellitus, thyroid disorders, kidney and liver disease or malignancies), pregnancy and breastfeeding. Additionally, all the female RA patients participated in Polish National Health Fund Therapeutic Programs employing TNF-blockers, i.e., B.33: “Treatment of aggressive forms of rheumatoid arthritis (RA) and juvenile idiopathic arthritis (JIA)” (03.0000.333.02) or B.45: “Treatment of an aggressive form of rheumatoid arthritis (03.0000.345.02)”. Anti-TNF-α treatment was administered over a 15-month period. Treatment was given as recommended by the manufacturers—for adalimumab 40 mg subcutaneously (SC) every other week, for etanercept 50 mg SC every week, for certolizumab pegol 400 mg SC at weeks 0, 2 and 4, followed by 200 mg every 2 weeks thereafter and for golimumab 50 mg SC, once a month. All of the female RA patients were continuing current anti-rheumatic therapy, including methotrexate at a stable dose (25 mg/week) and prednisone (≤7.5 mg/day). Furthermore, all subjects were receiving daily 5 mg dose of folic acid. Concomitant medications were kept unchanged for the duration of the study. Baseline characteristics of patients are summarized in Table 1.

At the study baseline and 3, 9 and 15 months after starting anti-TNF-α therapy, the effectiveness of TNFαI treatment was assessed by means of the DAS28 indicator calculated based on the number of swollen and tender joints from among the 28 joints included, erythrocyte sedimentation rate (ESR) and the patient’s global assessment of disease activity on a 100 mm visual analog scale (VAS). Furthermore, patients were submitted at each visit to laboratory tests, such as: complete blood count, inflammation markers—including ESR and plasma concentrations of C-reactive protein (CRP), creatinine and liver enzymes. The changes in clinical characteristics that occurred during the 15-month TNFαI therapy are summarized in Table 2. Patients who did not experience an adequate response to treatment were excluded from the study. Adequate response to treatment was defined—in accordance with principles of the Polish National Health Fund Therapeutic Programs—as reduction in DAS28 by a value greater than 1.2 after the first three months of TNF-α inhibitor therapy and further reduction in DAS28 by 1.2 recorded during subsequent medical examinations performed 9 and 15 months after administration of the first TNFαI dose.

Twenty-six age-matched healthy female volunteers from the Medical University of Silesia in Katowice, Poland were investigated as controls. Subjects were selected on the basis of their medical history, clinical examination and laboratory screening. All volunteers enrolled in this study had not suffered from any diseases that required hospitalization and had not undergone any surgical procedures during the preceding 3 years. Furthermore, the results of their routine laboratory tests (i.e., complete blood count, ESR, plasma fasting glucose, fasting lipid profile, creatinine, liver enzymes, rheumatoid factor (RF) and CRP) were within the reference range. Subjects were excluded from the study if they had been taking steroidal or non-steroidal anti-inflammatory drugs. None of the volunteers were cigarette smokers or had any history of drug or alcohol abuse. We selected women maintaining a healthy body weight and having a body mass index (BMI) < 25 kg/m^2^.

According to the assay manufacturers’ recommendations, we used serum instead of plasma for evaluation of all aggrecan turnover biomarkers. Samples of venous blood were drawn between 7.00 and 9.00 am after overnight fasting and were clotted for 30 min and then separated by means of centrifugation. Serum aliquots obtained from healthy subjects and RA patients were immediately stored in the temperature of −80 °C until the time of assay.

During the whole investigation period we were following the guidelines and regulations of the Helsinki Declaration in 1975, as revised in 1983. The Ethical Committee of the Medical University of Silesia in Katowice (KNW/0022/KB/56/I/12/13) approved the research protocol used in this study. All healthy volunteers and RA patients provided their written informed consent.

### 2.2. Serologic and Inflammatory Parameters

Measurement of RF and CRP (normal value < 5 mg/L) was performed at Konelab Prime 30ISE, bioMérieux, Craponne, France. RF was considered positive when the concentration measured was higher than the cut-off value of 15 U/mL. Additionally, we tested for anti-CCP antibodies by using an enzyme-linked immunosorbent assay (ELISA) kit (Euroimmun, Lubeck, Germany). The level of anti-CCP antibodies was considered positive when the concentration was higher than the cut-off value of 5 U/mL. ESR (normal range for women: 3–12 mm/h) was determined using the Westergren method (Sediplus^®^ S2000, Sarstedt, Germany).

### 2.3. Immunoassay of CS846 Epitope and AGC

Serum CS846 levels were determined in duplicate by a competitive enzyme-linked immunosorbent assay from IBEX Pharmaceuticals, Inc. (Montreal, QC, Canada). This ELISA assay with specific IgM monoclonal antibody recognizes an epitope present on the largest newly synthesized aggrecan molecules and absent from smaller degraded fragments that can still aggregate with hyaluronan. In brief, all serum samples were diluted 1:5 before assay. The minimal detectable level of CS846 was 7 ng/mL. All samples were tested within 1 day in order to eliminate the effects of inter-assay variation. The intra-assay coefficient of variation (CV) was <6%.

AGC concentrations in serum samples were analyzed by ELISA kit from Cloud-Clone Corp. (Katy, TX, USA), using antibodies recognizing epitopes in the CS-rich region of the aggrecan core protein. The assay was performed as per the manufacturer’s protocol. The analytical sensitivity was less than 0.59 ng/mL. All samples were tested in 1 day, thus inter-assay variation was insignificant. The intra-assay CV was <10%.

### 2.4. Immunoassay of ADAMTS-4, ADAMTS-5 and TIMP-3

Analysis of serum concentrations of ADAMTS-4, ADAMTS-5, and TIMP-3 was based on a quantitative sandwich ELISA (Cloud Clone Corp., Katy, TX, USA), according to the manufacturer’s instructions. The samples for ADAMTS-4/-5 and TIMP-3 determination were diluted 1:10 and 1:5, respectively. The minimum detectable concentration was estimated to be <0.115 ng/mL for ADAMTS-4, <0.127 ng/mL for ADAMTS-5 and <0.056 ng/mL for TIMP-3. Testing of all samples was completed in 1 day to eliminate the effects of inter-assay variation. The intra-assay CVs were <10% for all analyzed parameters.

### 2.5. Statistical Analysis

Data analyses were performed using TIBCO Software, Inc. (2017) (Palo Alto, CA, USA). The Shapiro–Wilk test was used in order to verify the normality of the distribution. Data not normally distributed was log-transformed before the analyses. Variables are summarized as mean ± SD (for normal distribution) or median and interquartile (25th–75th percentile) range (for abnormal distribution). Variance homogeneity was assessed using Levene’s test. Data were evaluated using a repeated measures analysis of variance (RM-ANOVA) (normal distributed data) with a sphericity check by means of Mauchly’s test of sphericity or using the RM-ANOVA Friedman’s test (non-normal data). Post hoc analyses carried out in the case of significant differences between subgroups were based on the Tukey’s test (*p* value < 0.05) or the Mann–Whitney U-test (*p* value obtained after applying the Bonferroni correction, *p* < 0.05; six possible comparisons). Spearman’s rank correlation coefficient was used to evaluate the relationship between aggrecanases/TIMP-3 ratios and indicators of disease activity as well as biomarkers of aggrecan turnover in female RA patients. The significance in case of multiple comparisons was assessed against a reference *p* value obtained after applying the Bonferroni correction (*p* < 0.05; seven possible comparisons).

## 3. Results

### 3.1. Clinical Response

Out of a total of 50 female RA patients recruited for the study, 31 patients (62%) completed 15 months of anti-TNF-α treatment, while 19 (38%) discontinued TNFαI and were excluded from our analysis. Among the patients excluded, TNFαI were discontinued due to the following reasons: no response (5 patients), loss of response (3 patients), intolerance (3 patients), surgical procedures (4 patients) and withdrawal of consent to participation in the therapy (4 patients). Overall, thirty-one female RA patients who continued the TNFαI therapy for 15 months were included in our analysis and presented in this study.

During the treatment with TNFαI, a significant clinical improvement was noted in all RA patients. According to the EULAR response criteria, 31 (100%) patients were good responders at 3 months [25] and this effect was sustained up to the 15th month. The clinical parameters, including number of tender and swollen joints, VAS, DAS28 and biologic parameters (ESR and CRP), decreased in all patients following the anti-TNF-α treatment (Table 2).

### 3.2. Serum Concentrations of CS846 and AGC

**Figure 2:**
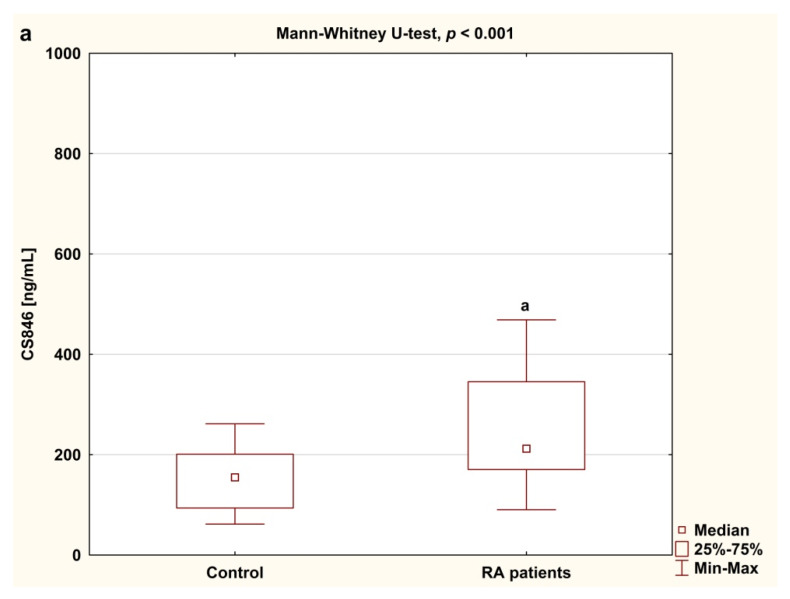

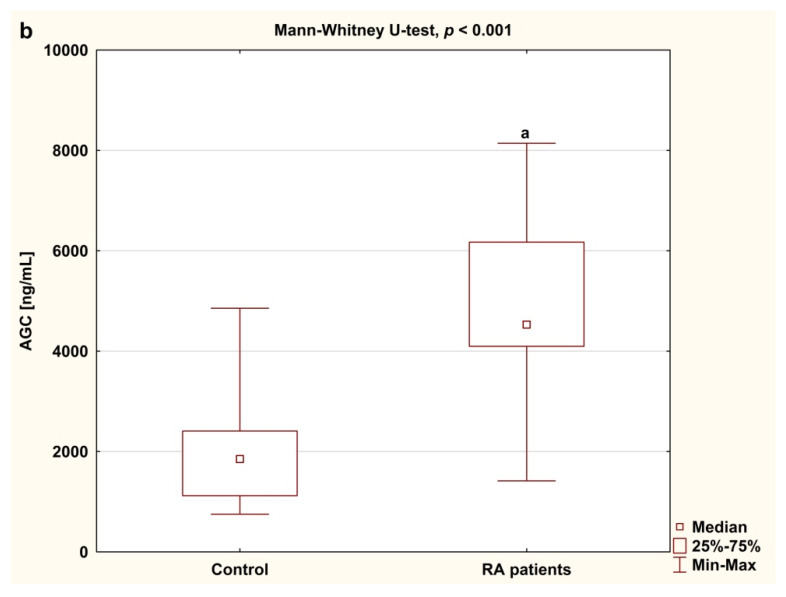
Serum levels of CS846 (**a**) and AGC (**b**) in RA patients before anti-TNF-α therapy and in controls. Results expressed as median and interquartile (25th–75th percentile) range. Data analyzed using Mann–Whitney U test. ^a^ statistically significant differences compared to controls. AGC, aggrecan fragments; anti-TNF-α, anti-tumor necrosis factor α; CS846, chondroitin sulfate epitope 846; RA, rheumatoid arthritis.

**Figure 3:**
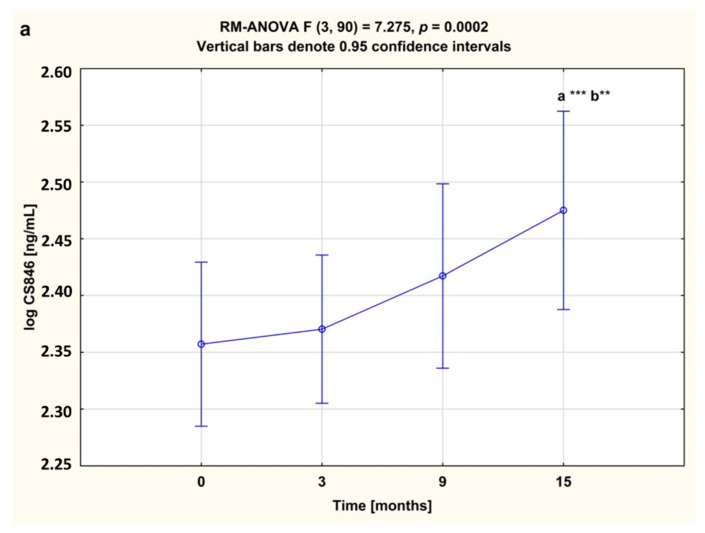

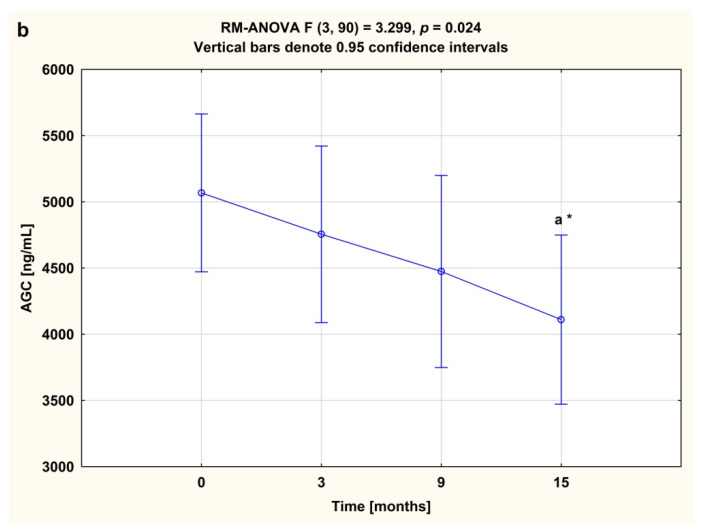
Temporal course of serum CS846 (**a**) and AGC (**b**) levels in RA patients during 15-month anti-TNF-α therapy. Results expressed as mean (standard deviation, SD). Data not normally distributed (CS846) was log-transformed prior to analyses. Data analyzed using one-way repeated measures analysis of variance (RM-ANOVA), followed by Tukey’s multiple comparisons test. ^a^ statistically significant differences compared to baseline; ^b^ statistically significant differences compared to 3 months after therapy. * *p* < 0.05; ** *p* < 0.01; *** *p* < 0.001. AGC, aggrecan fragments; CS846, chondroitin sulfate epitope 846.

Among the biomarkers analyzed, serum levels of cartilage formation marker CS846 epitope and cartilage destruction marker AGC were higher in RA women before anti-TNF-α therapy than in healthy subjects (both *p* < 0.001; Figure 2a,b). Levels of CS-846 were significantly higher compared to baseline, whereas levels of AGC were significantly lower in serum of RA patients during 15 month of anti-TNF therapy (*p* < 0.001 and *p* < 0.05, respectively; Figure 3a,b).

### 3.3. Serum Concentrations of ADAMTS and TIMP-3

**Figure 4:**
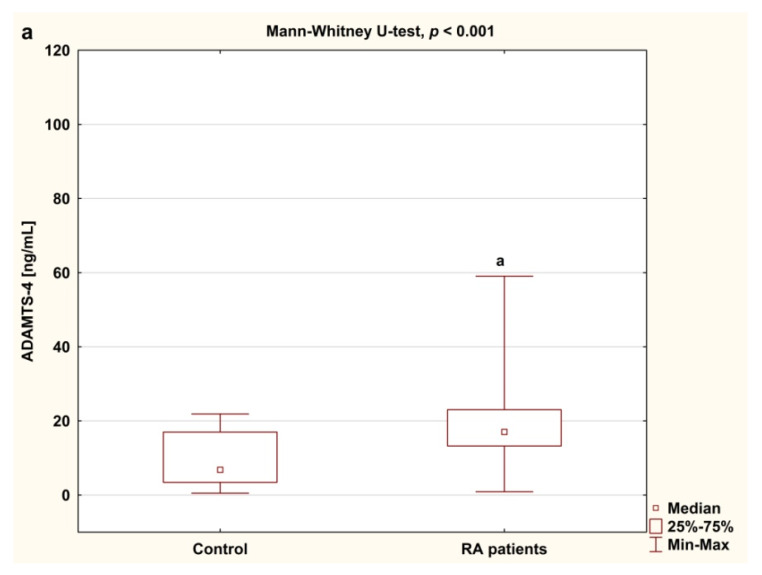

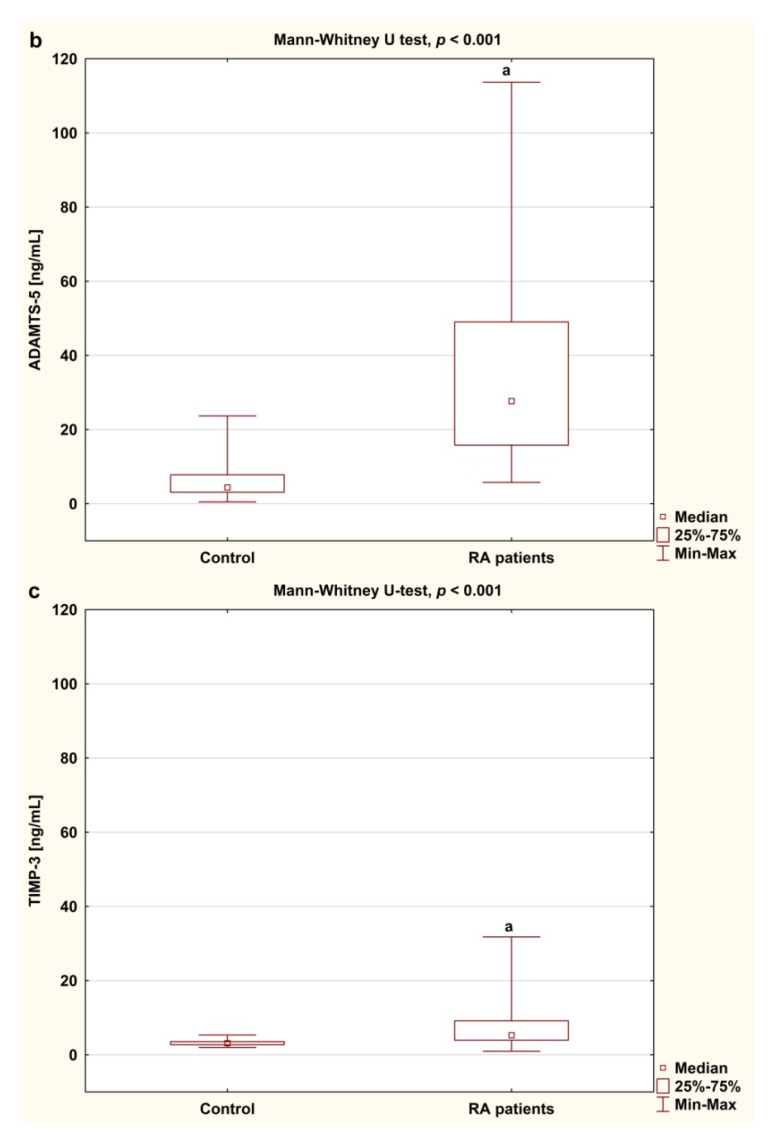
Serum levels of ADAMTS-4 (**a**), ADAMTS-5 (**b**) and TIMP-3 (**c**) in RA patients and in controls. Results expressed as median and interquartile (25th–75th percentile) range. Data analyzed using Mann–Whitney U test. ^a^ statistically significant differences compared to controls. ADAMTS-4 and ADAMTS-5, disintegrin and metalloproteinase with thrombospondin motifs 4 and 5; RA, rheumatoid arthritis; TIMP-3, tissue inhibitor of metalloproteinase-3.

The concentrations of serum ADAMTS-4, ADAMTS-5 and TIMP-3 in female RA patients participating in biologic therapy and in healthy individuals are presented in Figure 4a–c. Before the treatment with TNFαI, serum levels of ADAMTS-4, ADAMTS-5 and TIMP-3 were significantly higher in RA women than in healthy subjects (*p* < 0.001 for all comparisons; Figure 4a–c). The calculated ratio of ADAMTS-5 to TIMP-3 was significantly higher in RA patients than in healthy subjects, whereas the ratio of ADAMTS-4 to TIMP-3 did not differ from that in controls (Table 3).

**Figure 5:**
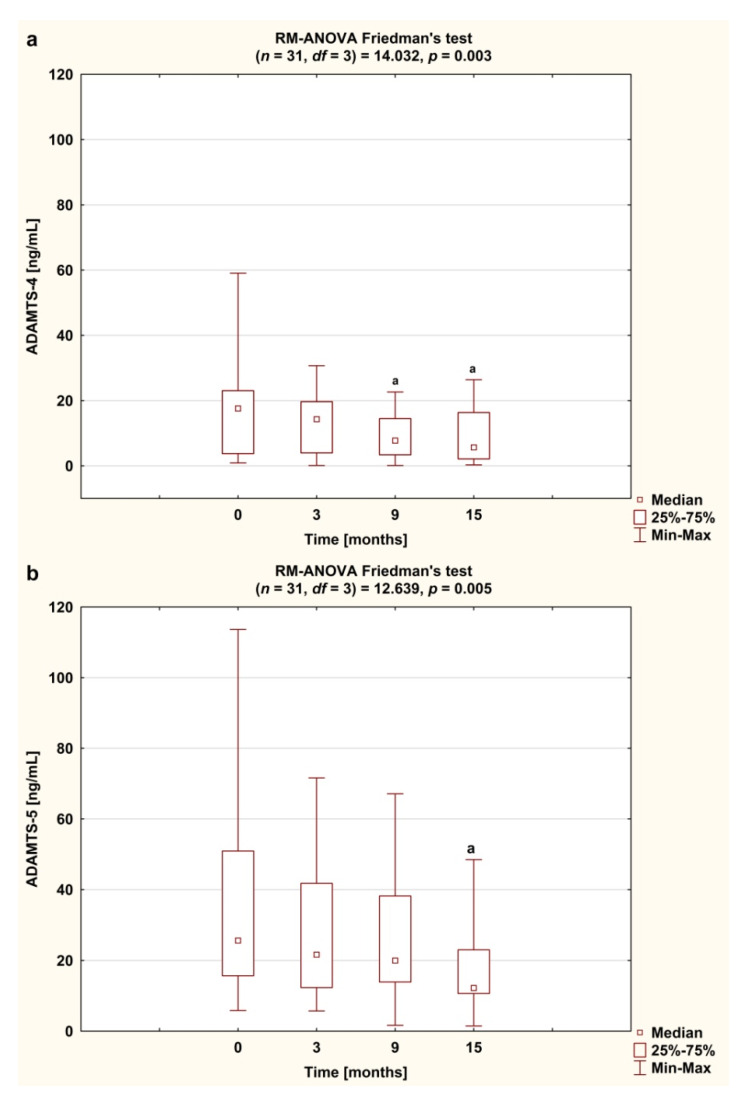

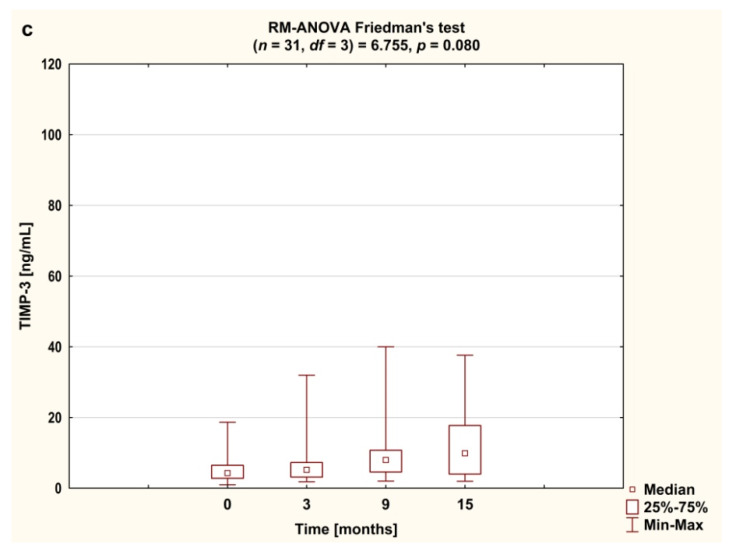
Temporal course of serum ADAMTS-4 (**a**), ADAMTS-5 (**b**) and TIMP-3 (**c**) levels in RA patients during 15-month anti-TNF-α therapy. Results expressed as median and interquartile (25th–75th percentile) range. Data analyzed using one-way repeated measures analysis of variance (RM-ANOVA) Friedman’s test. Differences noted for all variables considered significant at *p* < 0.0083 by applying Bonferroni correction. ^a^ statistically significant differences compared to baseline. ADAMTS-4 and ADAMTS-5, disintegrin and metalloproteinase with thrombospondin motifs 4 and 5; anti-TNF-α, anti-tumor necrosis factor α; RA, rheumatoid arthritis; TIMP-3, tissue inhibitor of metalloproteinase-3.

Serum concentration of ADAMTS-4 in RA patients was downregulated following anti-TNF-α therapy (*p* < 0.0083; Figure 5a). The lowest concentration of ADAMTS-4 was noticed after 15 months of treatment (*p* < 0.0083; Figure 5a). Similarly, serum concentration of ADAMTS-5 decreased significantly in response to the TNFαI therapy (*p* < 0.0083; Figure 5b). As shown in Table 4, these changes were accompanied by significantly reduced ADAMTS-4/TIMP-3 ratio and ADAMTS-5/TIMP-3 ratio. In contrast, serum levels of TMIP-3 were not affected by the treatment (*p* = 0.080; Figure 5c).

The analysis of the relationship between ADAMTS/TIMP-3 ratios and clinical (SJC, TJC, DAS28) and laboratory (ESR, CRP) indicators of disease activity as well as biomarkers of aggrecan turnover (CS846, AGC) in female RA patients at baseline and after 15 months of anti-TNF-α treatment are presented in Table 5. However, no significant correlations were found between ADAMTS/TIMP-3 ratios and any of the parameters evaluated.

### 3.4. Serum Concentrations of CS846 and AGC and ADAMTS/TIMP-3 Ratios Depending on the Type of TNF-α Inhibitor Used

**Figure 6:**
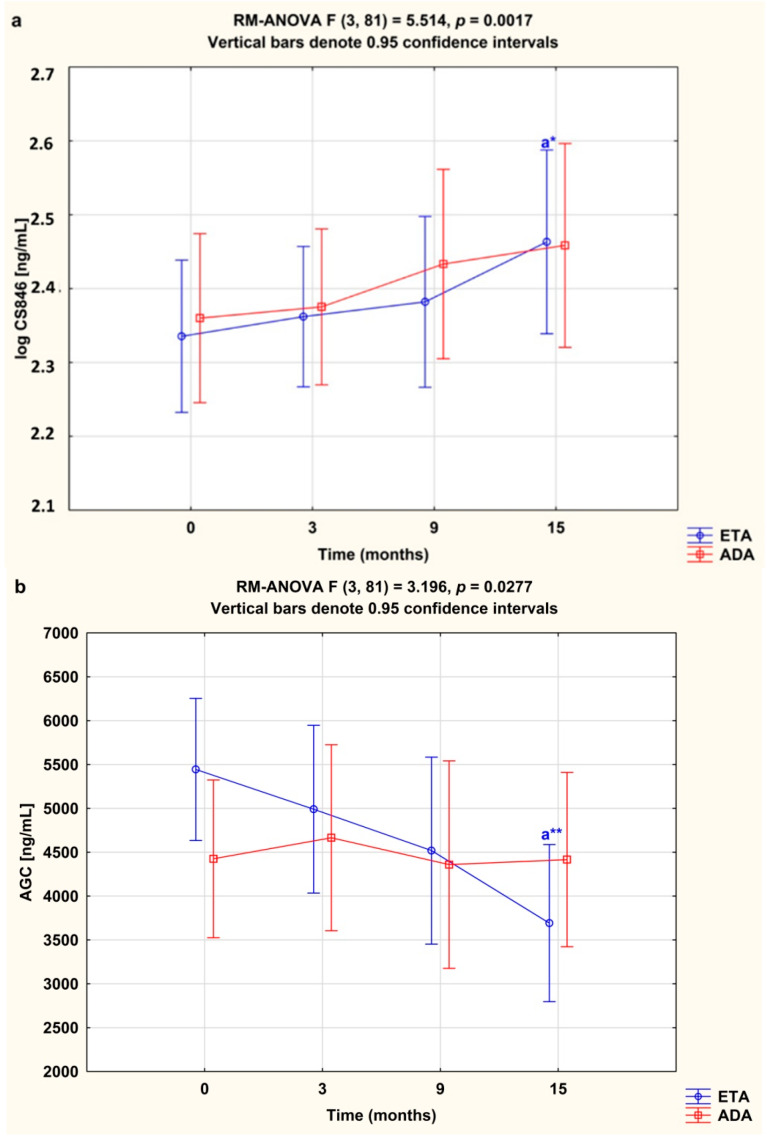
Temporal course of serum CS846 (**a**) and AGC (**b**) levels in RA patients during 15-month therapy with either etanercept (*n* = 16) or adalimumab (*n* = 13). Results expressed as mean (standard deviation, SD). Data not normally distributed (CS846) was log-transformed prior to analyses. Data analyzed using repeated measures analysis of variance (RM-ANOVA), followed by Turkey’s multiple comparisons test. ^a^ statistically significant differences compared to baseline. * *p* < 0.05; ** *p* < 0.01. AGC, aggrecan fragments; ADA, adalimumab; CS846, chondroitin sulfate epitope 846; ETA, etanercept.

In our study we wanted to check whether different types of TNF-α inhibitors may influence the levels of aggrecan turnover markers in RA patients. We compared the changes in levels of serum CS846 and AGC in female RA patients who completed a 15-month anti-TNF-α treatment with either etanercept or adalimumab. Administration of etanercept led to a significant increase in serum levels of CS846 epitope, and a decrease in serum levels of AGC in RA patients after 15 months of therapy (*p* < 0.05 and *p* < 0.01, respectively; Figure 6a,b). On the other hand, there were no significant changes in serum levels of CS846 and AGC following treatment with adalimumab (*p* = 0.784 and *p* = 0.800, respectively; Figure 6a,b).

**Figure 7:**
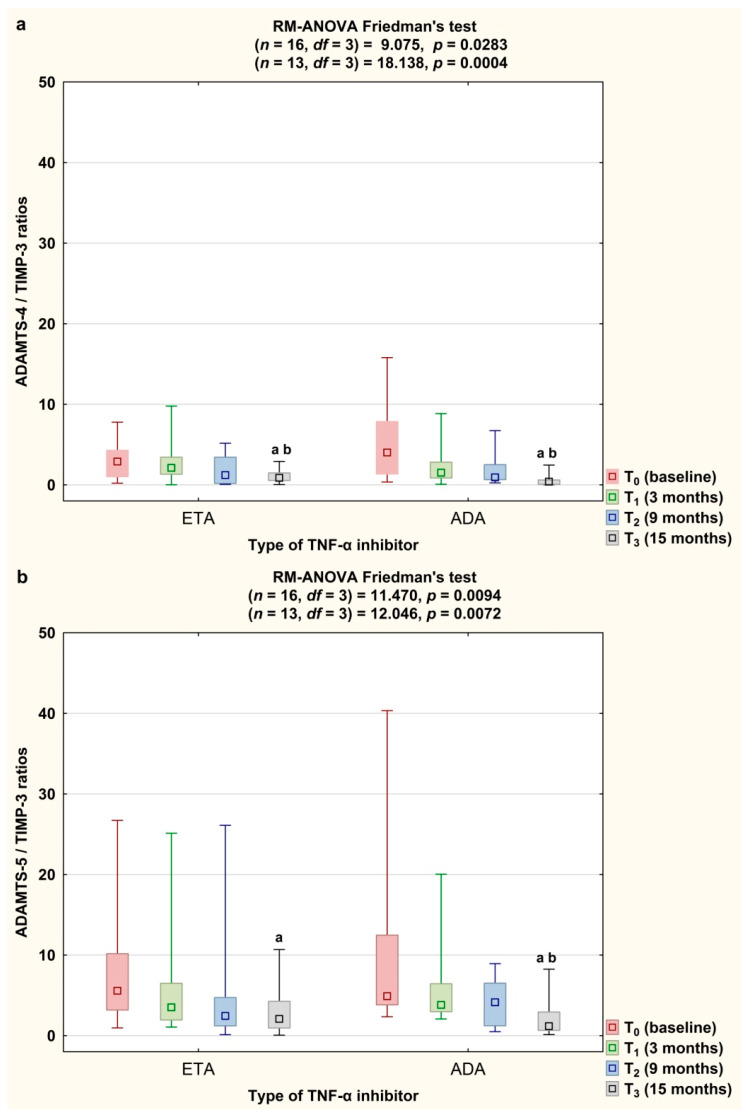
Temporal course of ADAMTS-4 to TIMP-3 ratios (**a**) and ADAMTS-5 to TIMP-3 ratios (**b**) in RA patients during 15-month therapy with either etanercept (*n* = 16) or adalimumab (*n* = 13). Results expressed as median and interquartile (25th–75th percentile) range and minimum–maximum. Data analyzed using repeated measures analysis of variance (RM-ANOVA) Friedman’s test. Differences noted for all variables considered significant at *p* < 0.0083 by applying Bonferroni correction. ^a^ statistically significant differences compared to baseline, ^b^ statistically significant differences compared to 3 months after therapy. ADA, adalimumab; ADAMTS-4 and ADAMTS-5, disintegrin and metalloproteinase with thrombospondin motifs 4 and 5; ETA, etanercept; RA, rheumatoid arthritis; TIMP-3, tissue inhibitor of metalloproteinase-3.

We also demonstrated that therapy with both etanercept and adalimumab decreased significantly the ADAMTS-4/TIMP-3 ratio (*p* < 0.0083 and *p* < 0.001, respectively; Figure 7a). The ADAMTS-5/TIMP-3 ratio decreased in both etanercept- and adalimumab-treated RA patients as well (*p* < 0.0083 and *p* < 0.001, respectively; Figure 7b).

## 4. Discussion

Prevention of early depletion of aggrecan in RA patients may contribute to slowing the progression of the disease and therefore to improved joint function. Our study demonstrates that effective 15-month anti-inflammatory treatment with TNFαI was associated with improvement of aggrecan turnover, assessed through serum levels of aggrecan-derived synthesis and degradation molecules (CS846 and AGC, respectively) and ADAMTS levels in female RA patients. Indeed, higher levels of the CS846 epitope and lower levels of AGC were noted under anti-TNF-α treatment. We also found that etanercept therapy could be associated with a better improvement concerning CS846 and AGC than adalimumab therapy. To the best of our knowledge, no data regarding the effects of anti-TNF-α treatment on biochemical markers of aggrecan turnover in patients with RA has been published so far. Moreover, we have expanded our previous results regarding positive changes in extracellular matrix remodeling in RA patients treated with TNFαI [26].

Since the 846 epitope is expressed only on the largest, newly synthesized aggrecan molecules, it may be suggested that the increased levels of CS846 that we found in all RA patients treated with TNFαI may be a reparative response of the chondrocytes. Several studies revealed that the release of the CS846 epitope, normally present in fetal or juvenile articular cartilage, but almost completely absent from mature adult cartilage, correlates positively with aggrecan synthesis [20,27]. Consistently with our results, the increased concentrations of CS846 in patients with chronic RA were described previously [27,28]. Interestingly, the CS846 levels appear to be suppressed in the serum of patients with early, rapidly progressing RA [28]. Moreover, Manson et al. [28] found an inverse linear correlation between serum CS846 level in patients with RA and inflammatory markers, such as ESR and CRP. Our findings are in accordance with these data, since the decrease in disease activity of RA paralleled the increase in a marker of aggrecan synthesis, the CS846 epitope, in all RA patients following the anti-TNF-α treatment. This may suggest an indirect relationship between cartilage repair and inflammation. It is well known that TNF-α and IL-1β, two components which play active roles in perpetuating inflammation in RA, are powerful inhibitors of aggrecan synthesis [4,21,28]. Thus, the very low, often subnormal, levels of serum CS846 noted by Manson et al. [28] in RA patients with active joint destruction may indicate an inhibition of aggrecan synthesis or changes in the processes leading to CS846 release from tissues into body fluids. Because the consequence of rapidly progressing RA is a significant or even complete loss of articular cartilage, the release of matrix components decreases and interpretation of very low levels of CS846 may be difficult [29,30]. To summarize, increased levels of CS846 in RA patients following anti-TNF-α therapy may reflect the ability or at least an attempt to repair the damaged cartilage matrix resulting from effective disease control and suppression of inflammation. Previous studies have shown that TNF-α blockade in RA diminishes local inflammation in the joints by decreasing synovial cell infiltration as well as the expression of adhesion molecules, chemokines and cytokines, which coincides with reduction in acute phase reactants, such as CRP and IL-6 [31,32]. Consistently with our results, Maksymowych et al. [33] demonstrated that effective treatment of ankylosing spondylitis patients with etanercept is associated with an increase in serum levels of CS846 and a reduction in serum levels of a neoepitope generated through cleavage of type II collagen by collagenases (C2C), suggesting increased synthesis and decreased degradation of cartilage matrix components, respectively.

We also showed that the circulating levels of aggrecan fragments were greater in female RA patients before anti-TNF-α treatment when compared to age-matched healthy individuals. High levels of aggrecan fragments were previously found in synovial fluid from patients with RA, OA and after knee injury [30,34,35,36]. Furthermore, it was also found that patients with early RA who had high levels of aggrecan fragments in synovial fluid progressed more rapidly towards joint destruction than patients with low levels of the mentioned fragments [30]. Our findings differ from those of Rousseau et al. [37], who demonstrated that the total levels of aggrecan fragments carrying the G1 and/or G2 domains were lower in serum of RA patients than in healthy controls. These discrepancies can result from methodological differences, especially with regard to the specificity of antibodies used in enzyme immunoassay, capable of recognizing aggrecan fragments of different sizes and clearance. In addition, these divergences may be caused in part by differences in sex of the patients, disease activity, disease duration and the type of anti-rheumatic drugs used.

Our study has also demonstrated that blocking TNF-α represents a therapy that may protect the structure of articular cartilage by inhibiting aggrecan degradation via the aggrecanases. Since specific aggrecanase-cleaved fragments of aggrecan were found in synovial fluid from patients with RA and OA, it was suggested that both ADAMTS-4 and ADAMTS-5 are the main mediators responsible for aggrecan cleavage in the early events of cartilage remodeling under pathological conditions [9,11]. High expression of both enzymes was identified in cartilage and synovial tissue or blood of patients with RA and OA [15,38,39,40]. In our study, both serum ADAMTS-4 and ADAMTS-5 levels were greater in female RA patients before anti-TNF-α therapy in comparison to the healthy individuals. Furthermore, we confirmed that circulating levels of aggrecan fragments and ADAMTS-4 and ADAMTS-5 levels were significantly downregulated following 15-month anti-TNF-α therapy. Lack of studies regarding this issue makes it impossible to compare the obtained results with those of other authors. However, decreased ADAMTS-4 expression in human synoviocytes was noted after etanercept treatment [41]. Considering our results, we can suppose that decreased serum levels of both aggrecanases during anti-TNF therapy may be related to the ability of TNFαI to promote programmed cell death through the inhibition of nuclear factor-κB activation. As reported earlier, TNF-α blockade induces apoptosis of synovial fluid monocytes/macrophages, which are a constant source of proteases such as ADAMTS and MMPs, thus protecting against early aggrecan loss and cartilage erosion [15,31,32].

Several investigators suggest that the accelerated breakdown of cartilage matrix in RA may be caused by the quantitative imbalance between ADAMTS and TIMP-3. The last one, unlike other TIMPs, is the most potent tissue inhibitor of ADAMTS-4 and ADAMTS-5 [9,42]. There is less knowledge about the changes in circulating TIMP-3 level. Elevated TIMP-3 expression was reported in human rheumatoid and osteoarthritic synoviocytes [42]. In the present study, the levels of TIMP-3 increased in serum of female RA patients before anti-TNF-α therapy, possibly reflecting an endogenous corrective response to increased levels of active ADAMTS. This suggestion is supported by increased aggrecanases/TIMP-3 ratios, especially in the case of ADAMTS-5 in RA women when compared to the healthy individuals. Furthermore, during anti-TNF-α therapy, the ratios of both ADAMTS to TIMP-3 decreased. The results obtained indicate that the balance between TIMP-3 and ADAMTS synthesis during RA is disturbed in favor of aggrecanases, which promotes aggrecan catabolism. Thus, significant reduction both in ADAMTS levels and in ADAMTS/TIMP-3 ratios under anti-TNF-α treatment may represent an important mechanism for preventing future development of joint damage in RA patients.

Because the degradation of the aggrecan core protein is also associated with the action of matrix metalloproteinases, we can suppose that the decreased levels of serum aggrecan fragments at 15 months after initiation of TNFαI therapy may result from reduced activity of these enzymes. As reported previously for infliximab and etanercept, RA patients receiving adalimumab or certolizumab pegol showed significantly reduced levels of serum MMP-1 and MMP-3 [43,44,45,46]. In addition to excessive activity of proteases, oxidative stress induced by reactive oxygen species (ROS) is also involved in cartilage matrix destruction in RA. It is well known that ROS may act at different levels of the cartilage degradation process by inhibiting ECM formation and/or inducing oxidative matrix damage and regulating the expression of MMPs and ADAMTS [47,48]. Thus, it seems that the reduction of serum AGC level in RA women that we found in our study may be caused in part by reduced intracellular ROS production. Anti-TNF-α therapy has indeed been shown to suppress ROS generation in RA patients. It is also reported that TNF-α blocking agents reduce serum reactive oxygen metabolite levels and oxygen stress marker levels [49,50,51].

## 5. Conclusions

In conclusion, we report for the first time that prolonged anti-TNF-α therapy combined with MTX, not only provides rapid clinical response, but also has a beneficial effect on aggrecan turnover. Thus, the increase in serum levels of the CS846 epitope with significant reduction of the AGC, ADAMTS-4 and ADAMTS-5 levels and ADAMTS/TIMP-3 ratios under anti-TNF-α treatment indicates a shift of aggrecan turnover towards its synthesis, which may represent an important mechanism for preventing future joint damage and disability in RA patients.

Furthermore, regarding the effects of each TNF-α inhibitor on the levels of aggrecan turnover biomarkers, i.e., CS846 and AGC, in female RA patients, our results demonstrate the superiority of etanercept therapy in preventing cartilage damage when compared to adalimumab. However, further studies are necessary to confirm our results definitively, given the relatively small number of patients in studied groups.

## Figures and Tables

**Table 1:** Baseline characteristics of female RA patients treated with TNF-α inhibitors.

Characteristic	Value
All women with RA, *n* (%)	50 (100)
Age (years), mean (SD)	47.52 (11.91)
Disease duration (years), median (IQR)	6 (3–12)
Height (cm), mean (SD)	163.58 (6.78)
Weight (kg), mean (SD)	65.52 (14.40)
BMI (kg/m^2^), mean (SD)	24.46 (5.17)
RF positive, *n* (%)	44 (88)
Anti-CCP positive, *n* (%)	43 (86)
SJC 28, median (IQR)	7 (5–10)
TJC 28, median (IQR)	12 (9–14)
VAS, median (IQR)	80 (70–80)
DAS 28-ESR, mean (SD)	5.83 (0.49)
ESR (mm/h), median (IQR)	17.0 (10.0–29.0)
CRP (mg/L), median (IQR)	6.37 (3.0–10.30)
Anti-rheumatic therapy, *n* (%)	
Methotrexate (25 mg/week)	50 (100)
Prednisone (≤ 7.5 mg/day)	50 (100)
Folic acid (5 mg/day), *n* (%)	50 (100)
TNFαI therapy, *n* (%)	
Etanercept (Enbrel)	24 (48)
Adalimumab (Humira)	22 (44)
Certolizumab pegol (Cimzia)	2 (4)
Golimumab (Simponi)	2 (4)

Data are presented as mean (standard deviation, SD) or median, inter-quartile (25th–75th percentile) range or percentage (%). anti-CCP, anti-cyclic citrullinated peptide antibody; BMI, body mass index; CRP, C-reactive protein; DAS 28-ESR, disease activity score based on the evaluation of 28 joints; ESR, erythrocyte sedimentation rate; IQR, inter-quartile range; RA, rheumatoid arthritis; RF, rheumatoid factor; SJC 28, swollen joint count of 28 joints; TJC 28, tender joint count of 28 joints; TNF-α, tumor necrosis factor α; TNFαI, tumor necrosis factor α inhibitors; VAS, visual analog scale.

**Table 2:** Time-course changes in biochemical, clinical and functional measures during 15-month anti-TNF-α therapy.

	Time after Starting Anti-TNF-α Therapy			
	T_0_ (Baseline)	T_1_ (3 Months)	T_2_ (9 Months)	T_3_ (15 Months)
Women with RA, *n* (%)	31 (100)			
Age (years), mean (SD)	45.87 (12.28)			
Disease duration (years), median (IQR)	5 (3–11)			
Growth (cm), mean (SD)	163.77 (6.63)			
Weight (kg), mean (SD)	65.89 (14.60)			
BMI (kg/m^2^), mean (SD)	24.62 (5.65)			
RF positive, *n* (%)	28 (90.32)			
Anti-CCP positive, *n* (%)	26 (83.87)			
SJC 28, median (IQR)	7 (5–10)	2 (0–3) ^a, c^	0 (0–0) ^a, b^	0 (0–0) ^a, b^
TJC 28, median (IQR)	12 (9–16)	4 (2–7) ^a, c^	1 (0–2) ^a, b^	0 (0–0) ^a, b, c^
VAS, median (IQR)	80 (80–80)	40 (30–50) ^a, c^	20 (10–30) ^a, b^	15 (5–20) ^a, b^
DAS 28-ESR, median (IQR)	5.78 (5.51–6.24)	3.92 (3.08–4.42) ^a, c^	2.75 (2.24–3.13) ^a, b^	2.19 (1.75–2.51) ^a, b, c^
Disease activity, *n* (%)				
High (>5.1)	31 (100)	2 (6.45)	0 (0)	0 (0)
Moderate (>3.2 and ≤5.1)	0 (0)	20 (64.52)	3 (9.68)	0 (0)
Low (≤3.2 and >2.6)	0 (0)	4 (12.91)	14 (45.16)	5 (16.13)
Remission (≤2.6)	0 (0)	5 (16.13)	14 (45.16)	26 (83.87)
ESR (mm/h), median (IQR)	17.0 (10.0–34.0)	14.0 (9.0–23.0)	13.0 (9.0–18.0) ^a^	13.0 (8.0–18.0) ^a^
CRP (mg/L), median (IQR)	6.3 (3.08–14.0)	4.0 (2.0–9.0)	4.0 (2.0–4.3) ^a^	4.0 (1.5–5.1) ^a^
TNFαI therapy, *n* (%)				
Etanercept (Enbrel)	16 (51.62)			
Adalimumab (Humira)	13 (41.93)			
Certolizumab pegol (Cimzia)	2 (6.45)			

Data are presented as mean (standard deviation, SD) or median, inter-quartile (25th–75th percentile) range or percentage (%). Data analyzed using one-way repeated measures analysis of variance (RM-ANOVA) Friedman’s test. Differences noted for all variables considered significant at *p* < 0.0083 by applying Bonferroni correction. ^a^ statistically significant differences compared to T_0_; ^b^ statistically significant differences compared to T_1_; ^c^ statistically significant differences compared to T_2_. anti-CCP, anti-cyclic citrullinated peptide antibody; anti-TNF-α, anti-tumor necrosis factor α; BMI, body mass index; CRP, C-reactive protein; DAS 28-ESR, disease activity score based on the evaluation of 28 joints; ESR, erythrocyte sedimentation rate; IQR, inter-quartile range; RA, rheumatoid arthritis; RF, rheumatoid factor; SJC 28, swollen joint count of 28 joints; TJC 28, tender joint count of 28 joints; TNFαI, tumor necrosis factor α inhibitors; VAS, visual analog scale.

**Table 3:** Concentration ratios of ADAMTS to TIMP-3 in RA patients before anti-TNF-α therapy and in controls.

Characteristic	Control A	RA Patients (*n* = 50) B	*p* A vs. B
ADAMTS-4/TIMP-3	1.99 (1.27–2.67)	2.86 (1.29–4.57)	NS
ADAMTS-5/TIMP-3	1.34 (1.04–1.92)	4.45 (2.61–8.40)	*p* < 0.001

Data are presented as median, inter-quartile (25th–75th percentile) range. Data analyzed using Mann–Whitney U test. NS differences not significant. ADAMTS-4 and ADAMTS-5, disintegrin and metalloproteinase with thrombospondin motifs 4 and 5; anti-TNF-α, anti-tumor necrosis factor α; RA, rheumatoid arthritis; TIMP-3, tissue inhibitor of metalloproteinase-3.

**Table 4:** Concentration ratios of ADAMTS to TIMP-3 in RA patients before and during 15-month anti-TNF-α therapy.

Characteristic	RA Patients (*n* = 31)				*p*					
	Time after Starting Anti-TNF-α Therapy									
	T_0_ (baseline) A	T_1_ (3 months) B	T_2_ (9 months) C	T_3_ (15 months) D	A vs. B	A vs. C	A vs. D	B vs. C	B vs. D	C vs. D
ADAMTS-4/TIMP-3	3.11 (1.18–6.21)	2.06 (0.96–3.57)	0.79 (0.56–1.51)	0.76 (0.17–2.22)	NS	<0.001	<0.001	<0.0083	<0.0083	NS
ADAMTS-5/TIMP-3	5.01 (3.79–12.48)	3.73 (2.72–6.55)	2.63 (1.23–5.64)	1.74 (0.84–3.96)	NS	<0.0083	<0.001	NS	<0.001	NS

Data are presented as median, inter-quartile (25th–75th percentile) range. Data analyzed using one-way repeated measures analysis of variance (RM-ANOVA) Friedman’s test. Differences noted for all variables considered significant at *p* < 0.0083 by applying Bonferroni correction. NS differences not significant. ADAMTS-4 and ADAMTS-5, disintegrin and metalloproteinase with thrombospondin motifs 4 and 5; anti-TNF-α, anti-tumor necrosis factor α; RA, rheumatoid arthritis; TIMP-3, tissue inhibitor of metalloproteinase-3.

**Table 5:** The relationship between ADAMTS/TIMP-3 ratios and clinical (SJC, TJC, DAS28) and laboratory (ESR, CRP) indicators of disease activity as well as biomarkers of aggrecan turnover (CS846, AGC) in RA patients at baseline and after 15 months of anti-TNF-α treatment.

Characteristic	RA Patients (*n* = 31) Time after Starting Anti-TNF-α Therapy			
	T_0_ (Baseline)		T_3_ (15 Months)	
	ADAMTS-4/TIMP-3	ADAMTS-5/TIMP-3	ADAMTS-4/TIMP-3	ADAMTS-5/TIMP-3
SJC 28	−0.338 ^NS^	0.150 ^NS^	0.171 ^NS^	−0.171 ^NS^
TJC 28	0.029 ^NS^	−0.328 ^NS^	0.211 ^NS^	−0.167 ^NS^
DAS 28−ESR	−0.369 ^NS^	−0.144 ^NS^	0.138 ^NS^	0.074 ^NS^
CRP	−0.062 ^NS^	−0.183 ^NS^	0.305 ^NS^	0.463 ^NS^
ESR	−0.054 ^NS^	−0.187 ^NS^	−0.054 ^NS^	0.274 ^NS^
CS846	−0.059 ^NS^	0.256 ^NS^	−0.199 ^NS^	0.084 ^NS^
AGC	0.154 ^NS^	−0.054 ^NS^	0.405 ^NS^	0.231 ^NS^

Data expressed as *r* values (correlation coefficient) according to Spearman rank correlation. Correlations were considered significant at: *p* < 0.007 by applying a Bonferroni correction; NS differences not significant. ADAMTS-4 and ADAMTS-5, disintegrin and metalloproteinase with thrombospondin motifs 4 and 5; AGC, aggrecan fragments; anti-TNF-α, anti-tumor necrosis factor α; CRP, C-reactive protein; CS846, chondroitin sulfate epitope 846; DAS 28-ESR, disease activity score based on the evaluation of 28 joints; ESR, erythrocyte sedimentation rate; RA, rheumatoid arthritis; SJC 28, swollen joint count of 28 joints; TJC 28, tender joint count of 28 joints; TIMP-3, tissue inhibitor of metalloproteinase-3.
